# Impact of Physical Rehabilitation on Endometriosis and Adenomyosis-Related Symptoms: A Systematic Review and Meta-Analysis

**DOI:** 10.3390/jcm14238284

**Published:** 2025-11-21

**Authors:** Ángel Rodríguez-Ruiz, Beatriz Sierra-Artal, Mario Lozano-Lozano, Francisco Artacho-Cordón

**Affiliations:** 1 Department of Radiology and Physical Medicine, University of Granada, Av. Doctor Jesús Candel Fábregas, 11, 18016 Granada, Spainfartacho@ugr.es (F.A.-C.); 2 Physical Therapy Center ‘Enrique Sierra’, 50015 Zaragoza, Spain; 3 Biohealth Research Institute in Granada (ibs.GRANADA), 18012 Granada, Spain; 4 Department of Physiotherapy, University of Granada, Avda. de la Ilustración, 60, 18006 Granada, Spain; 5 Sport and Health University Research Institute (iMUDS), University of Granada, 18071 Granada, Spain; 6 Consorcio de Investigación Biomédica en Red (CIBER) of Epidemiology and Public Health (CIBERESP), 28029 Madrid, Spain

**Keywords:** endometriosis, adenomyosis, physical rehabilitation, pain, quality of life, exercise, electrotherapy

## Abstract

**Objectives:** The aim of this study is to summarize recent evidence of the effectiveness of rehabilitation interventions in managing symptoms related to endometriosis and adenomyosis. **Methods:** The review protocol was registered previously (CRD42022236516). A systematic search was conducted in the Medline, Web of Science, and Scopus databases for studies published up to 23 July 2025 that reported the effects of any rehabilitation intervention in women diagnosed with endometriosis or adenomyosis. Risk of bias was assessed, and meta-analyses were performed. **Results:** A total of 970 studies were identified, of which 19 reports from 17 trials met the inclusion criteria. Approximately one-third of the trials focused on electrophysical agents, another third on exercise programs, and the remaining studies included manual therapy-based interventions—such as pelvic floor physiotherapy (PFP), or Swedish massage—as well as other modalities. Most trials assessed changes in pain, quality of life (QoL), and mental health, showing consistent improvements following intervention. Additional outcomes evaluated included lumbopelvic impairments, sexual function, and bone mineral density. Meta-analyses of eleven studies on pain and five on QoL revealed significant effects, favoring the intervention groups. **Conclusions:** This review highlights promising benefits of physical rehabilitation, particularly in patients with endometriosis. A range of approaches—including therapeutic exercise, electrophysical agents, and PFP—may contribute to improvements in endometriosis-related clinical outcomes, especially pain and QoL.

## 1. Introduction

Endometriosis and adenomyosis are among the most common benign gynecological diseases in women on reproductive age, although endometriosis is being acknowledged to be more than a gynecological disorder, affecting multiple organs [1]. While endometriosis is characterized by the development and growth of endometrial-like tissue outside the uterus [2], this ectopic tissue is located in the myometrium in the case of adenomyosis, where it elicits hyperplasia and hypertrophy in surrounding smooth muscle cells [3]. Regarding the prevalence of endometriosis, published estimates range from 1–2% [4] to 10% [5,6], with significant differences according to the diagnostic method or the choice of sampling framework [7]. The prevalence of adenomyosis varies widely, with an average rate of 20–25% [8], with nearly 30% of cases being asymptomatic [9] and most of the cases involving women of late reproductive age (40–50 years) [8].

In addition to similarities in pathogenesis, in which similar key molecular events are present [10] and deep endometriotic nodules have been suggested to be the consequence of a cervical or uterine adenomyotic pathology [11], the symptomatic burden is similar between endometriosis and adenomyosis. Hence, patients usually report pain in the pelvic region, which is usually exacerbated during menstruation (dysmenorrhea) or some activities of daily life such as defecation (dyschezia), urination (dysuria), or sexual intercourse (dyspareunia) [12]. Moreover, these conditions are strongly associated with elevated infertility rates [13,14], psychological alterations [15,16], and physical impairments [17,18]. As a consequence, endometriosis and adenomyosis can have a crucial impact on work productivity and quality of life (QoL) [15,16,19], causing a substantial economic burden for health systems [15,20]. Medical and surgical treatments (designed to inhibit the growth of the endometriotic implants and to remove or destroy the endometriotic implants, respectively) are usually prescribed. Nevertheless, a considerable proportion of patients do not fully respond to conventional treatments [21], which often fail to address the multifactorial nature of these conditions. Factors such as central sensitization and pelvic floor dysfunctions may contribute to the persistence and chronification of symptoms, highlighting the need for complementary approaches—such as physical rehabilitation and pelvic floor physiotherapy—that target these additional mechanisms.

Because of this need, there is emerging interest in the potential therapeutic effectiveness of alternative approaches to the clinical management of symptoms. In this context, rehabilitation, through the vast array of therapeutic strategies for pain control, might offer a substantial benefit to ameliorate pain-related disabilities and to improve QoL in these women. For instance, therapeutic exercise, cognitive behavioral therapy, electrotherapy, or massage, among others, have demonstrated significant improvements in pain, function, and quality of life in patients with a variety of chronic pain conditions, and, therefore, they are promising avenues for research in endometriosis and adenomyosis [22]. Thus, the aim of this systematic review is to summarize the recent evidence of the effectiveness of rehabilitation strategies for the management of endometriosis- and/or adenomyosis-related symptoms.

## 2. Materials and Methods

### 2.1. Protocol and Registration

Details of the protocol for this systematic review were registered on the International Prospective Registry of Systematic Reviews (PROSPERO) (CRD42022236516).

This systematic review was conducted in accordance with the Preferred Reporting Items for Systematic Reviews and Meta-Analyses (PRISMA) guidelines [23]. According to PRISMA guidelines, the specific question that this review aimed to answer was “Could be considered rehabilitation approaches for the clinical management of endometriosis- and adenomyosis-related symptoms?”.

### 2.2. Search Strategy and Eligibility Criteria

The Medline (via PubMed searcher), Web of Science (WoS), and Scopus databases were used to search for published studies reporting on effect of any rehabilitation intervention in women with endometriosis and/or adenomyosis. The last search was performed on 23 July 2025. The detailed search strategy is displayed in Appendix A (Appendix A.1, Appendix A.2 and Appendix A.3). No restrictions were placed during the database search.

Inclusion criteria for this systematic review were as follows: studies published in English or Spanish, controlled trials, studies focusing on patients with endometriosis and/or adenomyosis exclusively (or at least with stratified analyses for these gynecological disorders), and studies evaluating the effect of any intervention that could be applied in a physical rehabilitation care unit.

Although randomized controlled trials (RCTs) are considered the gold standard for evaluating the effectiveness of interventions, this systematic review also includes controlled non-randomized studies, such as retrospective studies with a control group. The rationale for this decision is based on the limited availability of high-quality RCTs addressing physical rehabilitation interventions in women with endometriosis and/or adenomyosis. Including controlled observational studies allows for a broader understanding of the potential benefits of these interventions, especially in under-researched areas.

Two independent researchers (B.S.A. and A.R.R.) performed the selection of the studies using Covidence systematic review software (version 5.0.131, Covidence.org, Veritas Health Innovation, Melbourne, Australia), screened the retrieved studies, and assessed the methodological quality of the selected studies. F.A.C. resolved disagreements in the screening process.

### 2.3. Data Extraction, and Quality and Certainty of Evidence Assessment

The research team developed a standardized template to extract the key data from each study, including the following: authors, year of publication, target condition (endometriosis and/or adenomyosis), country, study design, number of participants, type of intervention in both experimental and control groups, characteristics of the interventions, outcome assessments (measured time points, domains, and instruments), and a summary of the intervention effects. Given the wide variety of interventions addressed, the data were organized into separate tables according to the nature of the intervention: (i) electrophysical agents, (ii) therapeutic exercise, and (iii) manual therapy and other approaches. Data extraction was independently performed by two researchers (B.S.A. and A.R.R.).

For quality assessment, the Revised Cochrane Risk of Bias tool for randomized trials (RoB 2) was used. This framework is designed to evaluate the risk of bias in randomized trials using a domain-based approach, focusing on five key areas: the randomization process, deviations from intended interventions, missing outcome data, outcome measurement, and selection of the reported result. Each domain includes signaling questions that guide the assessment, with judgments categorized as “low risk,” “some concerns,” or “high risk” of bias. This structured methodology ensures a comprehensive and systematic evaluation, enhancing the validity and reliability of systematic reviews and meta-analyses. RoB 2 emphasizes transparency and methodological rigor, enabling researchers to critically appraise the evidence and draw informed conclusions about the efficacy and safety of healthcare interventions [24].

The GRADE system (Grading of Recommendations, Assessment, Development, and Evaluation) was applied to assess the strength of recommendations and the quality of evidence for each outcome. GRADE classifies the quality of evidence into four levels—high, moderate, low, or very low—based on factors such as study design, risk of bias, consistency of results, directness of evidence, precision, and potential publication bias [25].

### 2.4. Statistical Analysis

A quantitative analysis of the data was conducted (M.L.L.). Based on the included studies and the availability of relevant data, SPSS version 30.0 (IBM Corp., Chicago, IL, USA) was used to perform meta-analyses of rehabilitative interventions that assessed pain using a 0–10 scale (NRS or VAS) and QoL using the Endometriosis Health Profile (EHP) questionnaire. Only studies reporting complete data for pain and/or QoL and including a comparison with a control group were eligible for inclusion in the meta-analysis. Of the thirteen studies assessing pain and nine assessing QoL identified in the systematic review, eleven [26,27,28,29,30,31,32,33,34,35,36] and five [27,28,31,34,36], respectively, met the inclusion criteria for quantitative synthesis.

Data were extracted from the tables, figures, and/or the main text of the articles. For the meta-analysis, the standardized mean difference (SMD) between post-intervention and baseline scores, along with their corresponding standard deviations (SDs), was used. From these, effect sizes (Cohen’s d) and 95% confidence intervals (CIs) were calculated. Statistical significance was set at *p* < 0.05.

Heterogeneity was assessed using the I^2^ statistic, H^2^ statistic, and Tau-squared (τ^2^), which estimates between-study variance. Depending on the degree of heterogeneity, either fixed-effect or random-effects models were applied. An I^2^ value greater than 50% was considered indicative of substantial heterogeneity, in which case a random-effects model was used; otherwise, a fixed-effect model was applied.

Additionally, subgroup analyses were conducted based on the type of rehabilitative intervention, being either electrophysical agent-, therapeutic exercise-, or manual therapy-based interventions. To assess whether effect sizes differed significantly between subgroups, a test for between-group heterogeneity (Q statistic) was performed. Forest plots were generated to visually summarize the results of individual studies and subgroups.

## 3. Results

**Figure 1:**
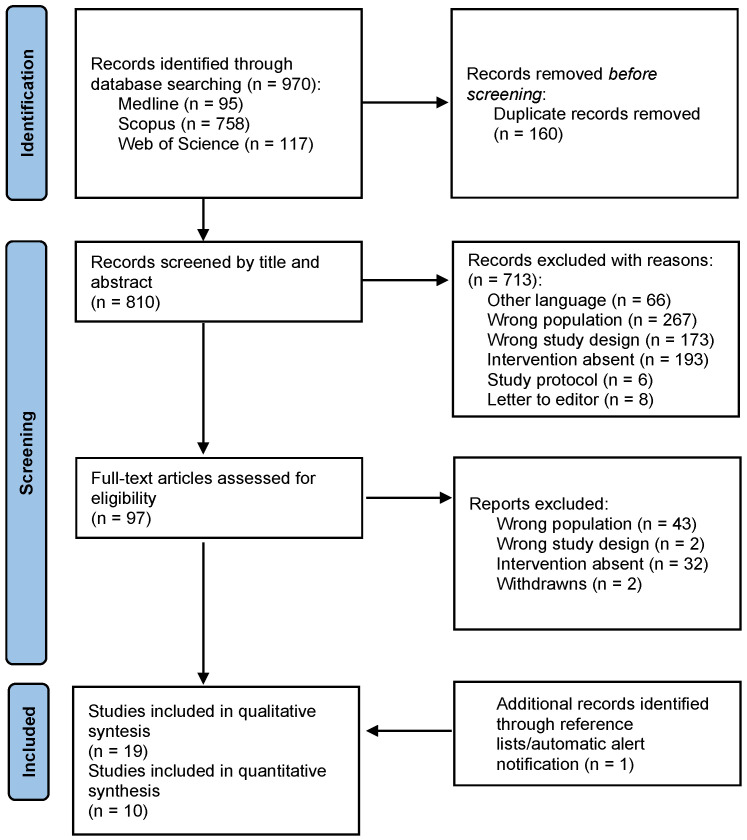
Flow diagram.

Figure 1 depicts the flow of studies throughout the selection process. A total of 970 records were retrieved (95 from Medline, 117 from WoS, and 758 from Scopus). After removing 160 duplicates, 810 records remained for title and abstract screening, of which 713 were excluded for not meeting the inclusion criteria. Of the 97 full-text articles assessed for eligibility, 79 were excluded because they did not evaluate any rehabilitation intervention in women with endometriosis or adenomyosis. Additionally, one relevant article identified through reference list screening met the inclusion criteria. In total, 19 reports from 17 controlled trials were included in this systematic review.

### 3.1. Characteristics of Studies

As shown in Table 1, all trials investigated the impact of rehabilitation interventions in patients with endometriosis, although five also included some women diagnosed with both endometriosis and adenomyosis. None of the trials specifically targeted women with adenomyosis alone.

Seven trials were conducted in European countries, five in the Americas, four in Asia, and one in Australia. Of the 19 reports included, 12 (63.2%) were published in the last five years (2020–2025), while 3 (15.8%) were published between 2015 and 2020 and 4 (21.1%) more than ten years ago. Most trials (76.5%) included fewer than 50 participants, with the smallest sample size being 15. In total, 849 women participated across the included trials.

Regarding diagnostic methods, endometriosis was confirmed via laparoscopy or laparotomy in six trials (35.3%), using medical imaging in three trials (17.6%), and based on symptoms in one trial (5.9%). Two trials included women diagnosed either by laparoscopy/laparotomy or imaging, and one trial included diagnoses based on laparoscopy/laparotomy or symptoms. In four trials (23.5%), the diagnostic method was not clearly reported. Similarly, ten out of seventeen trials (58.8%) did not report the disease stage.

### 3.2. Effectiveness of Rehabilitation in Women with Endometriosis/Adenomyosis

Table 2 summarizes the types of rehabilitation interventions evaluated, the outcomes assessed (including instruments used), and the main findings. A detailed description of each intervention is provided in Supplementary Table S1. As shown, a diverse range of trials investigated the effectiveness of rehabilitation strategies in patients with endometriosis and/or adenomyosis.

Six out of the seventeen included trials (35.3%) focused on electrophysical agents, such as transcutaneous electrical nerve stimulation (TENS), neuromuscular electrical stimulation (NMES), high-intensity laser therapy (HILT), radiofrequency, respiratory-gated auricular vagal afferent nerve stimulation (RAVANS), and transcranial direct current stimulation (tDCS) [27,29,32,33,34,37]. Another six trials (35.3%) evaluated therapeutic exercise-based programs, including progressive muscle relaxation (PMR), Hatha Yoga, and combinations of stretching, aerobic, resistance, and/or lumbopelvic stabilization exercises [28,30,36,38,39,40]. Three trials (17.6%) assessed interventions based on manual therapy—either alone [31], combined with therapeutic exercise [26,41], or with hydrotherapy [35]. Additionally, one trial investigated the effectiveness of self-care counseling and another explored virtual reality-based intervention [42,43].

Most studies assessed changes in pain, QoL, and/or mental health status. A smaller number also examined outcomes related to musculoskeletal impairments, cardiovascular parameters, sexual function, bone mineral density, or hormone levels.

**Table 1:** Descriptive synthesis of the included randomized controlled trials.

Authors	Year of Publication	Disease	Country	Study Design	Sample Size	Disease Stage	Disease Diagnosis
Del Forno et al., 2021 [26] Del Forno et al., 2023 [41]	2021 2023	ENDO + ADENO	Italy	RCT	ENDO (16); ENDO + ADENO (18)	IV (DIE)	US
Farshi et al., 2020 [42]	2020	ENDO	Iran	RCT	76	n.r.	Laparoscopy
Zhao et al., 2012 [38]	2012	ENDO	China	RCT	100	n.r.	Laparoscopy/laparotomy
Napadow et al., 2012 [37]	2012	ENDO	USA	crossover RCT	15	n.r.	Based on symptoms
Mira et al., 2020 [27]	2020	ENDO	Brazil	RCT	101	IV (DIE)	US or MRI
Bergström et al., 2005 [39]	2005	ENDO	Sweden	RCT	19	n.r.	Laparoscopy
Carpenter et al., 1995 [40]	1995	ENDO	USA	RCT	39	II: 36%; III: 26%; IV: 31%; n.r.: 7%	Laparoscopy
Gonçalves et al., 2016 [28]	2016	ENDO	Brazil	RCT	40	n.r.	n.r.
Thabet et al., 2018 [34]	2018	ENDO	Saudi Arabia	RCT	40	Mild/moderate endometriosis	Laparoscopy
Bi et al., 2018 [29]	2018	ENDO	China	Retrospective study	154	I: 9.1%; II: 29.9%; III: 40.3%; IV: 20.8%	Laparoscopy
Lutfi et al., 2023 [30]	2023	ENDO	Australia	RCT	22	n.r.	n.r.
Artacho-Cordón et al., 2023 [36] Salinas-Asensio et al., 2025 [44]	2023 2025	ENDO	Spain	RCT	31	I–III: 29%; IV: 71%	US/MRI/Laparoscopy
Muñoz-Gómez et al., 2023 [31]	2023	ENDO	Spain	RCT	41	n.r.	n.r.
Carralero-Martínez et al., 2022 [32]	2022	ENDO + ADENO	Spain	RCT	12	n.r.	n.r.
Merlot et al., 2022 [43]	2022	ENDO + ADENO	Canada	RCT	ENDO (34); ENDO + ADENO (11)	Superf. peritoneum(22.2%); DIE(75.6%); Other(2.2%)	MRI
Mechsner et al., 2023 [33]	2023	ENDO	Germany	RCT	36	n.r.	Laparoscopy or based on symptoms
Rodríguez-Ruiz et al., 2023 [35]	2024	ENDO	Spain	RCT	44	n.r.	US/MRI/Laparoscopy

ADENO: Adenomyosis; ENDO: Endometriosis; DIE: deep infiltrating endometriosis; MRI: magnetic resonance imaging; RCT: randomized controlled trial; US: ultrasound; USA: United States of America.

**Table 2:** (**a**) Summary of effectiveness of the electrophysical agent-based interventions on the randomized controlled trials included. (**b**) Summary of effectiveness of the therapeutic exercise-based interventions on the randomized controlled trials included. (**c**) Summary of effectiveness of the manual therapy-based interventions and others on the randomized controlled trials included.

(a) electrophysical agent-based interventions								
Authors	Intervention			Outcome				
	CG	IG	Duration, Frequency	Measured Time Point	Dimension		Instrument	Effect
Napadow et al., 2012 [37]	RAVANS or NVAS (n = 15)		RAVANS and NVAS in counterbalanced sessions ≥1 week apart.	At mid-point (15 min), post-intervention and 15 min post-intervention	Pain		Temporal summation	Reduced in the RAVANS session (*p* = 0.050).
							Cuff algometry	Reduced at all time points (*p* < 0.05), greater in RAVANS.
							DNIC	No significant effects or interaction (*p* > 0.100).
							VAS	No significant effects or interaction (*p* > 0.300).
					Anxiety		VrAS	Lower at all time points in RAVANS (*p* < 0.010), not in NVAS.
					Heart rate		ECG	No significant effects or interaction (*p* > 0.100).
					LF-HRV, HF-HRV, LF/HF		ECG	No significant effects or interaction (*p* > 0.100).
					Respiratory rate		ECG	No significant effects or interaction (*p* > 0.100).
Mira et al., 2020 [27]	Usual care (n = 48)	Usual care + TENS (n = 53)	Twice a day, 20 min per application, for 8 weeks.	Post-intervention	Pain	Chronic pelvic pain	VAS	⊕ IG vs. CG (mean change: −2.55 vs. −0.27, *p* < 0.001).
						Deep dyspareunia	DDS	No between-group differences. ⊕ IG more (Δ = −0.67, *p* < 0.001) vs. CG (Δ = −0.27, *p* = 0.016).
						Dyschezia	DDS	No between-group differences. ⊕ IG more (Δ = −1.79, *p* < 0.001) vs. CG (Δ = −0.92, *p* = 0.022).
						Dysuria	DDS	No between-groups differences. ⊕ IG (mean change: −0.56, *p* = 0.034).
						Pain during spotting	DDS	No between-groups differences.
					QoL		EHP-30	⊕ Greater in infertility in CG (Δ = −5.99 vs. −2.00, *p* = 0.047). ⊕ in both groups in some domains (greater in IG); ⊕ IG in additional domains.
					Sexual function		FSFI	⊕ IG vs. CG in desire, arousal, orgasm, satisfaction, lubrication, pain and total score; ⊕ CG in satisfaction.
Thabet et al., 2018 [34]	Sham HILT	Pulsed HILT	Three times per week for 8 weeks	Post-intervention	Pain		PPi graphic scale	⊕ IG vs. CG (77.3% vs. 37.3%; *p* < 0.001)
							PR scale	⊕ IG vs. CG (mean score: 3.25 vs. 1.80, *p* < 0.001)
					QoL		EHP-5	No baseline data. QoL significantly better in IG vs. CG (15.8 ± 3.7 vs. 16.3 ± 3.6; *p* < 0.001).
Bi et al., 2018 [29]	No intervention (n = 71)	NMES (n = 83)	30 min/session, once daily, 3 sessions weekly, 10 weeks.	Mid- and post-intervention	Pain		NRS	No mid-intervention effect. Post-intervention: ⊕ IG vs. CG (Δ = −2.9 vs. −0.6, *p* = 0.020).
							ESSS	No mid-intervention effect. Post-intervention: ⊕ IG more than CG (Δ = −2.5 vs. −0.5, *p* = 0.040).
					QoL		SF-36	No mid-intervention changes. Post-intervention: IG improved more in PhCS (Δ = 8.7 vs. 0.9, *p* < 0.010) and MCS (Δ = 7.9 vs. 0.6, *p* < 0.010).
					Adverse effects		Ad hoc questionnaire	Fewer adverse effects in IG vs. CG (1.4% vs. 9.6%, *p* = 0.040).
Carralero-Martínez et al., 2022 [32]	Sham group: Deactivated CRMRF + physiotherapy + pain education.	CRMRF group (n = 41): CRMRF + physiotherapy + pain education.	10 sessions; once a week.	Mid- and post-intervention	Pain		VAS	⊕ IG vs. CG post-intervention (mean change: −2.74 vs. −0.95, *p* = 0.002).
					QoL		SF-12	⊕ IG vs. CG post-intervention in physical domain (mean change: 4.70 vs. 1.33, *p* = 0.034) and physical functioning (mean change: 5.23 vs. 0.99, *p* = 0.037)
Mechsner et al., 2023 [33]	Sham group (n = 18)	Active tDCS group (n = 18)	10 weekdays over 2 weeks; 20 min sessions with tDCS (active or sham).	Post-session, post-intervention and 5–12 days post-intervention	Pain	PPTs	Algometry	Post-intervention: ⊕ IG vs. CG in right lower abdomen (Δ = 1.9 vs. 0.1, *p* < 0.001).
						Pain intensity	NRS	Post-intervention: ⊕ IG vs. CG (Δ = −1.9 vs. −0.7, *p* = 0.003). No follow-up effect.
						CTRP	PCS	No between-groups differences.
					Depression		BDI	No between-groups differences.
**(b) therapeutic exercise-based interventions**								
**Authors**	**Intervention**			**Outcome**				
	**CG**	**IG**	**Duration, Frequency**	**Measured Time Point**	**Dimension**		**Instrument**	**Effect**
Zhao et al., 2012 [38]	GnRH agonist therapy (n = 50)	GnRH agonist therapy + PMR training (n = 50)	24 sessions, 40 min each, 2×/week over 12 weeks	Post-intervention	Anxiety		STAI	⊕IG vs. CG for state (−16.4 vs. −6.7; *p* = 0.020) and trait anxiety (−11.6 vs. −3.4; *p* = 0.030)
					Depression		HADS	⊕ IG vs. CG were observed (mean change: −2.6 vs. −0.3; *p* = 0.040)
					QoL		SF-36	Both groups improvement after intervention.
Bergström et al., 2005 [39]	Groserilin (G) (n = 11)	Groserilin (G) + Physical training (PT) (n = 8)	(G) 6 months. (PT) 5×/week (2 × 30 min, 3 × 60 min), 12 months.	6 months and post-intervention	BMD		DEXA	Femoral neck: Reduced BMD in the CG vs. IG post-intervention (*p* = 0.029). Spine: No between-groups differences.
Carpenter et al., 1995 [40]	Danazol *	Danazol + exercise *	4 sessions/week, 40 min/session.	24 weeks	Hormone levels (testosterone, SHBG and estradiol)		Molecular analysis	Testosterone lower in IG vs. CG (62.5 ± 10.2 vs. 111.9 ± 17.5, *p* = 0.020). No group differences in free testosterone, SHBG, or estradiol.
					Side effects		Ad hoc questionnaire	3 discontinued in CG due to side effects; none in IG. More side effects reported in CG at 24 weeks. No statistical analysis.
					Weight gain		Ad hoc questionnaire	No between-groups differences were observed.
					Time to recurrence		Ad hoc questionnaire	IG: 16 months; CG: 11 months. No between-group analysis performed.
Gonçalves et al., 2016 [28]	Usual care (n = 12)	Hatha Yoga (n = 28)	2×/week, 2 h/session for 8 weeks.	Post-intervention	QoL		EHP-30	Post-intervention: ⊕ IG more than CG in work domain (Δ = −35.93 vs. −3.75, *p* = 0.027). Both groups improved overall.
					Pain		VAS	⊕ IG vs. CG in average pain score.
Lutfi et al., 2023 [30]	Usual Care (n = 6)	TH exercise (n = 8) or VR exercise (n = 8)	TH: 1 h supervised session. VR: 1 h unsupervised session.	2 days post-intervention	Acute pelvic pain		VAS	No between-groups differences.
Artacho-Cordón et al., 2023 [36] Salinas-Asensio et al., 2025 [44]	Usual Care (n = 15)	Physio-EndEA group (n = 16)	9-week program in two phases. Phase 1: 1 week, 1–2 sessions. Phase 2: twice-weekly 90 min sessions	Post-intervention and at 1yr	QoL		EHP-30	⊕ IG vs. CG in global status, pain, and emotional well-being post-intervention and at 1yr (all *p* < 0.050).
					Pain	PPTs	Algometry	Improvement in IG vs. CG post-intervention (significant in pelvic and lumbar points; trend in right infraumbilical and second metacarpal) and at 1yr (significant in left lumbar).
						Current pelvic pain	NRS	⊕ Close-to-significant IG vs. CG post-intervention (−1.63 vs. −0.07; *p* = 0.060). No at 1yr.
						Dysmenorrhea	NRS	⊕ IG vs. CG post-intervention (−1.25 vs. −0.07, *p* < 0.050).
						Dyspareunia	NRS	No between-groups effects at post-intervention nor 1yr.
						Dyschezia	NRS	⊕ Close-to-significant IG vs. CG post-intervention (−0.75 vs. 0.47; *p* = 0.060). No at 1yr.
						Dysuria	NRS	No between-groups effects at post-intervention nor 1yr.
						CTRP	PCS	⊕ IG vs. CG post-intervention (−9.10 vs. −0.42; *p* < 0.050) and at 1yr (−6.57 vs. 1.40; *p* < 0.050).
					Lumbopelvic strength	Flexors	Resistance test	⊕ IG vs. CG post-intervention (23.67 vs. −3.96, *p* < 0.050) and 1yr (25.13 vs. 0.66, *p* < 0.050)
						Extensors	Resistance test	⊕ IG vs. CG post-intervention (33.02 vs. 5.84, *p* < 0.050). No at 1yr.
					Lumbopelvic stability		Sharmann test	⊕ IG vs. CG post-intervention (1.56 vs. 0.13, *p* < 0.050). No at 1yr.
					Muscle architecture	TrA, IOb, EOb and MF thickness, and MF width	US	⊕ Close-to-significant IG vs. CG post-intervention for TrA thickness (0.05 vs. −0.01; *p* = 0.076) and significant for right lumbar MF width (0.38 vs. 0.12; *p* < 0.050). No for other muscles or at 1yr.
					Fatigue		PFS	⊕ IG vs. CG in global score post-intervention (−1.89 vs. 0.18) and at 1yr (−0.81 vs. 0.31), with significant effects across all subscales (all *p* < 0.050).
					Physical fitness	Cardiorespiratory fitness	6MWT	⊕ IG vs. CG post-intervention (46.31 vs. −13.23; *p* < 0.001) and at 1yr (47.23 vs. 14.08; *p* < 0.050).
						Strength	Back dynamometer	⊕ IG vs. CG post-intervention (13.60 vs. −2.35; *p* = 0.088) and at 1yr (5.34 vs. −0.89; *p* < 0.050).
							Hand dynamometer	No between-groups effects at post-intervention nor 1yr.
						Lumbar flexibility	Schöber test	No between-groups effects at post-intervention nor 1yr.
						Body balance	Flamingo test	No between-groups effects at post-intervention nor 1yr.
					Sleep quality		PSQI	No between-groups effects at post-intervention nor 1yr.
					Mental health	Anxiety	HADS	⊕ IG vs. CG post-intervention (−3.62 vs. −0.08; *p* = 0.019) and at 1yr (−2.08 vs. 0.85; *p* < 0.050).
						Depression	HADS	⊕ IG vs. CG post-intervention (−1.69 vs. 0.92; *p* < 0.001) and at 1yr (−0.85 vs. 0.08; *p* < 0.050).
					Digestive complaints		GIQLI	⊕ IG vs. CG post-intervention (−14.77 vs. −0.77; *p* = 0.002) and at 1yr (−11.15 vs. 1.46; *p* < 0.050).
					Sexual function		FSFI	No between-groups effects at post-intervention nor 1yr.
**(c) manual therapy-based interventions**								
**Authors**	**Intervention**			**Outcome**				
	**CG**	**IG**	**Duration,** **Frequency**	**Measured Time Point**	**Dimension**		**Instrument**	**Effect**
Del Forno et al., 2021 [26] Del Forno et al., 2023 [41]	No intervention (n = 13)	PFP (n = 17)	5 sessions (30 min each) on weeks 1, 3, 5, 8 and 11.	4 months after randomization	LHAv change		US	⊕ IG vs. CG (20.0% vs. −0.5%, *p* = 0.020)
					LHAr change		US	No between-groups differences.
					LHAm change		US	No between-groups differences.
					Pain	Superficial dyspareunia	NRS	⊕ IG vs. CG (median change: −3 vs. 0, *p* < 0.010).
						Deep dyspareunia	NRS	⊕ Borderline IG vs. CG (median change: −1 vs. 0, *p* = 0.070).
						Dysmenorrhea	NRS	No between-groups differences.
						Chronic pelvic pain	NRS	⊕ IG vs. CG (median change: 0 vs. 0, *p* = 0.010)
						Ovulatory pain	NRS	Borderline reduction in IG vs. CG (median change: 0 vs. 0, *p* = 0.070)
						Dysuria	NRS	No between-groups differences.
						Dyschezia	NRS	No between-groups differences.
					Lower urinary tracts symptoms		BFLUTS	No between-groups differences.
					Gastrointestinal complaints		KESS	No between-groups differences.
					Sexual function		FSFI	No between-groups differences.
Muñoz-Gómez et al., 2023 [31]	Placebo (n = 20)	Manual Therapy group (n = 21)	The interventions lasted for 8 weeks, with one session for 30 min every 15 days.	Post-intervention (T1), 1-month (T2) and six-month (T3) follow-ups	Pain (pelvic pain)		VAS	⊕ IG vs. CG at T1 (−1.67 vs. −0.05, *p* = 0.030) and T3 follow-up (−3.81 vs. −0.55, *p* < 0.001).
					Lumbar mobility		Schöber test (cm)	⊕ IG vs. CG at T1 (3.43 vs. 0.04), T2 (0.46 vs. 0.13) and T3 (0.32 vs. −0.92) (*p* < 0.050).
					QoL		EHP-30	⊕ IG vs. CG at T1 in pain domain (−26.51 vs. −15.37; *p* = 0.003).
							SF-36	⊕ IG vs. CG at T1 in bodily pain (15.43 vs. 4.10; *p* = 0.030).
					Depression		BDI	No between-groups differences.
					Anxiety		STAI	No between-groups differences.
					Patient perception of change		PGICS	No between-groups differences.
Rodríguez-Ruiz et al., 2023 [35]	No intervention (n = 21)	HAMMAM (n = 23)	4 weeks; 3 sessions, 14-day intervals; 1.5 h/session	Post-intervention	Pain	Pelvic pain	NRS	No between-groups differences.
						Dysmenorrhea	NRS	⊕ IG vs. CG post-intervention (−1.27 vs. −0.29, *p* < 0.050).
						Dyspareunia	NRS	⊕ IG vs. CG post-intervention (−1.53 vs. −0.29, *p* < 0.050).
						Dyschezia	NRS	No between-groups differences.
						Dysuria	NRS	No between-groups differences.
						Pain interference	BPI	No between-groups differences.
						CTRP	PCS	No between-groups differences.
						PPTs	Algometry	⊕ IG vs. CG post-intervention at all pelvic points (0.39 vs. −0.06; *p* < 0.050).
					Subjective well-being		EBS-20	No between-groups differences.
					QoL		EHP-30	No between-groups differences.
Farshi et al., 2020 [42]	No intervention (n = 38)	Self-care counseling (n = 38)	7 weekly sessions, 60–90 min each.	1 month after intervention	Depression		BDI	No between-groups differences.
					Anxiety		STAI	⊕ IG vs. CG for state (−9.7 vs. 3.9; *p* < 0.001) and trait anxiety (−7.9 vs. 3.9; *p* < 0.001).
					QoL		SF-36	⊕ IG vs. CG for PhCS (3.9 vs. −4.3; *p* < 0.001) and MCS (3.8 vs. −2.8, *p* < 0.001).
Merlot et al., 2022 [43]	2D-Sham group (n = 22)	VR-Endocare group (n = 23)	1 session	240min post-intervention	Pain	Pain intensity	NRS	⊕ IG vs. CG at T15, T30 and T45. No between-groups effects at T60-T240.
						Pain relief	5-point scale	Improvement in IG vs. CG at all follow-ups points (*p* < 0.050).

6MWT: 6-min walking test; BDI: Beck Depression Inventory; BFLUTS: Bristol Female Lower Urinary Tract Symptoms; BMD: Bone Mineral Density; BPI: Brief Pain Inventory; CG: Control Group; CRMRF: Capacitive Resistive Monopolar Radiofrequency; CTRP: Catastrophizing thoughts related to pain; DDS: Deep Dyspareunia Scale; DEXA: Dual-Energy X-ray Absorptiometry; DNIC: Diffuse Noxious Inhibitory Control; EBS-20: Subjective Well-being Scale; ECG: Electrocardiography; EHP-5: Endometriosis Health Profile-5; EHP-30: Endometriosis Health Profile-30; EOb: external oblique; ESSS: Endometriosis Symptom Severity Score; FSFI: Female Sexual Function Index; G: Groserilin; GIQLI: Gastrointestinal Quality of Life Index; GnRH-a: Gonadotropin-Releasing Hormone agonist; HADS: Hospital Anxiety and Depression Scale; HF: High-Frequency band; HILT: High-Intensity Laser Therapy; HRV: Heart Rate Variability; IG: Intervention Group; IOb: internal oblique; KESS: Knowles-Eccersley-Scott-Symptom; LF: Low-Frequency band; LHAm: Levator Hiatal Area on maximum contraction; LHAr: Levator Hiatal Area at rest; LHAv: Levator Hiatal Area during maximum Valsalva maneuver; MCS: Mental Component Summary; MF: multifidus; NMES: Neuromuscular Electrical Stimulation; NRS: Numerical Rating Scale; NVAS: Nonvagal Auricular Stimulation; PCS: Pain Catastrophizing Scale; PFP: Pelvic Floor Physiotherapy; PFS: Piper Fatigue Scale; PGICS: Patient Global Perception of Change Scale; PhCS: Physical Component Summary; PMR: Progressive Muscle Relaxation; PPi: Present Pain intensity; PPT: Pressure Pain Threshold; PSQI: Pittsburgh Sleep Quality Index; PT: Physical Training; QoL: quality of life; RAVANS: Respiratory-gated Auricular Vagal Afferent Nerve Stimulation; SF-12: 12-item Short-Form Health Survey; SF-36: 36-item Short-Form Health Survey; SHBG: Sex Hormone-Binding Globulin; STAI: State-Trait Anxiety Inventory; tDCS: Transcranial Direct Current Stimulation; TENS: Transcutaneous Electrical Nerve Stimulation; TH: Telehealth; TrA: transversus abdominis; US: Ultrasound imaging; VAS: Visual Analogue Scale; VR: Virtual Reality; VrAS: Verbal Analogue Scale; ⊕ Improvement. * Not reported the number of participants allocated to each group.

#### 3.2.1. Effectiveness on Pain-Related Outcomes

Pain was the most frequently investigated outcome among the included trials, assessed in 13 out of 17 trials (76.5%) [26,27,28,29,30,31,32,33,34,35,36,37,43] using a variety of instruments, including the Numeric Rating Scale (NRS), Visual Analogue Scale (VAS), algometry, the Deep Dyspareunia Scale (DDS), the Pain Relief (PR) scale, the Endometriosis Symptom Severity Score (ESSS), and the Pain Catastrophizing Scale (PCS). All studies reported improvements in pain-related outcomes in the intervention group, except for one trial evaluating the effectiveness of a single session of a Telehealth- or Virtual Reality-delivered exercise [30].

Among the trials assessing electrophysical agents, interventions such as TENS, NMES, HILT, tDCS, and capacitive resistive monopolar radiofrequency (CRMRF) demonstrated significant reductions in pain intensity [27,29,32,33,34]. Additionally, RAVANS and tDCS were associated with improvements in pressure pain thresholds (PPTs) measured via algometry [33,37].

Similarly, therapeutic exercise-based interventions—including Hatha Yoga and combinations of stretching, aerobic, and resistance exercises—also led to reductions in pain intensity in women with endometriosis [28,36]. Furthermore, therapeutic exercise was effective in improving both PPTs and pain-related catastrophizing thoughts [36].

All three trials evaluating manual therapy approaches—pelvic floor physiotherapy (PFP) [26], manual therapy [31], and Swedish massage combined with hydrotherapy [35]—reported significant reductions in pain intensity. One additional trial investigating a single session of virtual reality-based treatment also found a significant reduction in pain compared to the control group [43].

While all studies assessed the immediate effects of interventions on pain relief, only four trials explored longer-term outcomes. These indicated sustained improvements in pain intensity at 3–6 months following PFP [26], manual therapy [31], and self-care counseling [42]. Moreover, one study reported improvements in lumbar PPTs at 12 months following a multimodal therapeutic exercise program [36].

#### 3.2.2. Effectiveness on Quality of Life

QoL was assessed in ten out of seventeen trials (58.8%) [27,28,29,31,32,34,35,36,38,42] using validated instruments such as the Endometriosis Health Profile-30 (EHP-30), EHP-5, and the 36- or 12-item Short-Form Health Surveys (SF-36 and SF-12).

Four trials evaluated the impact of electrophysical agents—including TENS, NMES, HILT, and CRMRF—on QoL [27,29,32,34], all of which reported improvements in overall health status or specific domains, particularly those related to physical functioning.

Similarly, three trials investigated the effects of therapeutic exercise programs—such as PMR, Hatha Yoga, or combinations of stretching, aerobic, and resistance exercises—and found significant improvements in QoL among participants in the intervention groups [28,36,38].

In contrast, no significant improvements in QoL were observed in trials evaluating a manual therapy protocol [31], a combination of Swedish massage and hydrotherapy [35], or a self-care counseling intervention [42] when compared to control groups.

#### 3.2.3. Effectiveness on Mental Health Status

Five trials evaluated anxiety levels using instruments such as the Visual Rating Anxiety Scale (VrAS), the State-Trait Anxiety Inventory (STAI), and the Hospital Anxiety and Depression Scale (HADS) [31,37,38,42,44]. Four of these trials (80.0%)—which focused on therapeutic exercise [38,44], self-care counseling [42] and RAVANS [37]—reported significant improvements in anxiety symptoms in the intervention groups compared to controls.

Depressive symptoms were also assessed in five trials [31,33,38,42,44] using the Beck Depression Inventory (BDI) or the HADS. Significant improvements in depression scores were observed in participants who underwent PMR therapy [38] and multimodal therapeutic exercise [44]. However, no significant effects were found following other interventions [31,33,42].

#### 3.2.4. Effectiveness on Other Disease-Related Outcomes

Several trials explored additional outcomes beyond pain, quality of life, and mental health, particularly focusing on musculoskeletal, sexual, gastrointestinal, cardiovascular, and hormonal parameters.

Musculoskeletal impairments were addressed both locally and systemically. Regarding local impairments in the lumbopelvic region, one trial reported improvements in pelvic floor muscle function—specifically in the levator hiatal area at rest and during maximal contraction—following PFP [26]. A multimodal therapeutic exercise program demonstrated benefits in abdominal and lumbar muscle function, including increased muscle strength, greater thickness of the transversus abdominis and width of the lumbar multifidus, and enhanced lumbopelvic stability [36]. Additionally, lumbar mobility improved following a manual therapy intervention in another trial [31]. The trial based on a multimodal therapeutic exercise program also reported systemic musculoskeletal benefits, including improvements in cardiorespiratory fitness and reductions in fatigue [44].

Sexual and gastrointestinal functions were evaluated in three and two trials, respectively. A TENS-based intervention was effective in improving various aspects of sexual function—including desire, arousal, orgasm, satisfaction, and total score [27]—whereas no significant improvements were observed following multimodal therapeutic exercise [44] or PFP [41]. In contrast, gastrointestinal symptoms improved after a multimodal therapeutic exercise program [44], but not following a PFP intervention [41].

Other outcomes were assessed in individual studies. One crossover trial evaluated cardiovascular and respiratory parameters, finding no significant changes in respiratory rate, heart rate, or heart rate variability [37]. Another study found that physical exercise helped prevent bone mineral density loss in the femoral neck, although not in the spine [39]. Additionally, one trial reported a reduction in total testosterone levels following exercise, while no significant changes were observed in free testosterone, sex hormone-binding globulin, or estradiol levels [40].

#### 3.2.5. Quality Assessment and Quantitative Analysis

**Figure 2:**
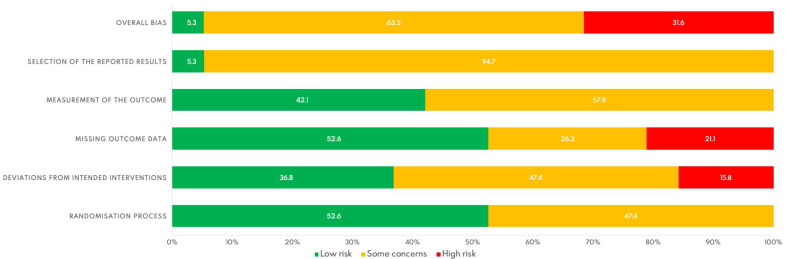
Risk of bias graph of included randomized controlled trials.

**Figure 3:**
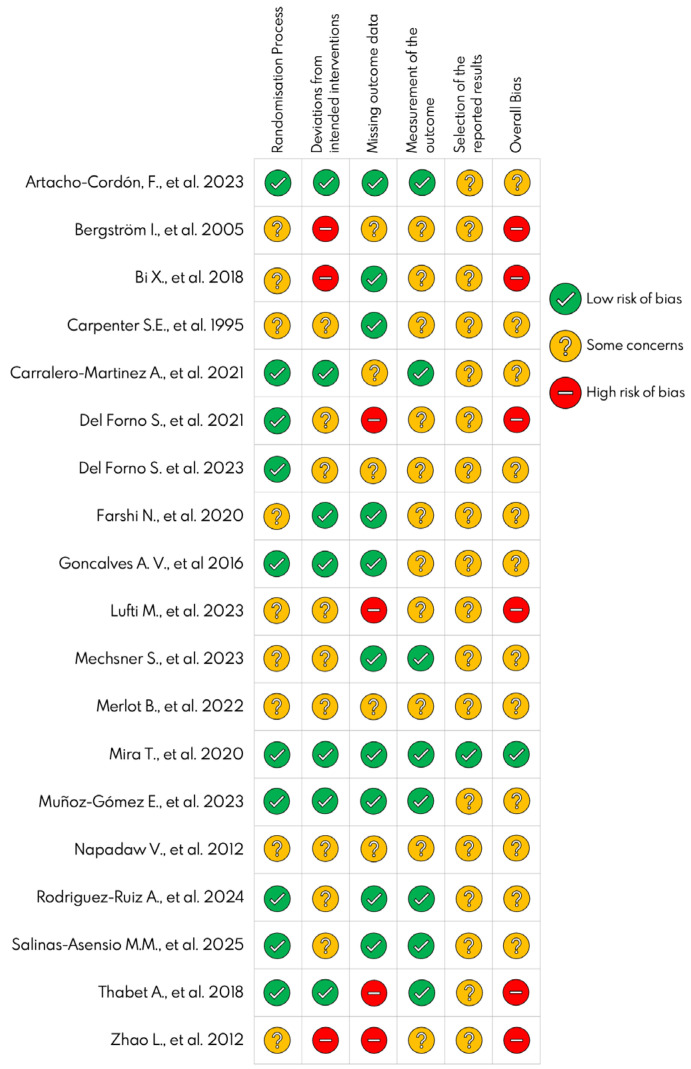
Risk of bias summary of the included randomized controlled trials [26,27,28,29,30,31,32,33,34,35,36,37,38,39,40,41,42,43,44].

The results of the risk of bias assessment are presented in Figure 2 and Figure 3. At the individual study level, only the trial conducted by Mira, Yela, Podgaec, Baracat, and Benetti-Pinto [27] was rated as having a low risk of bias across all domains. Overall, most studies were rated as having “some concerns” in the domains related to the measurement of the outcome and selection of the reported results, primarily due to the lack of a pre-specified statistical analysis plan. In the global assessment, 37.5% of the included studies were classified as having a high risk of bias.

In the analysis of the strength of recommendation and certainty of evidence according to the GRADE guidelines, the quality of evidence for pain intensity was rated as low when considering all interventions collectively, moderate for electrophysical agent-based treatments, low for manual therapy-based interventions, and very low for therapeutic exercise. Regarding quality of life (QoL), the evidence was rated as moderate when interventions were considered globally, low for both electrophysical and therapeutic exercise treatments, and very low for manual therapy-based interventions (Table 3).

**Figure 4:**
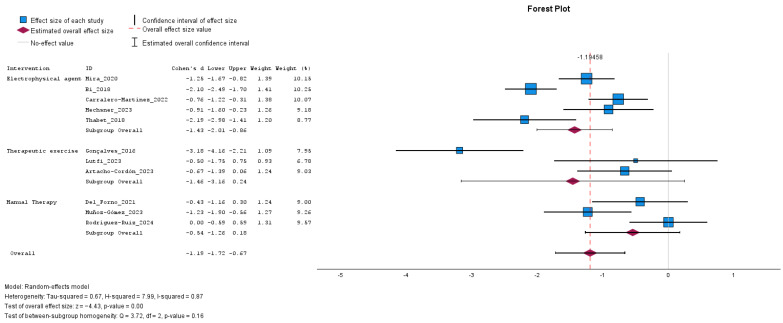
Forest plot presenting the effect of rehabilitative interventions on the improvement of pain intensity measured with a numeric scale in women with endometriosis compared with control; pre-post intervention data. Values on *x*-axis denote Cohen’s d. The diamond illustrates the 95% confidence interval of the pooled effects [26,27,28,29,30,31,32,33,34,35,36].

A total of eleven studies evaluating the effect of rehabilitative interventions on pelvic pain were included in the meta-analysis (Figure 4). The overall effect size indicated a significant reduction in pain in favor of the intervention group, with a mean difference of −1.19 (95% CI: from −1.72 to −0.67; *p* < 0.001). The effect was statistically significant for interventions based on electrophysical agents [−1.46 (95% CI: from −2.01 to −0.86; *p* < 0.001)] and approached significance for those based on therapeutic exercise [−1.46 (95% CI: from −3.16 to 0.24; *p* = 0.093)], while the effect of manual therapy was not statistically significant [−0.54 (95% CI: from −1.26 to 0.18; *p* = 0.139]. The analysis revealed substantial heterogeneity among the included studies (τ^2^ = 0.67, H^2^ = 7.99, and I^2^ = 87%), indicating considerable variability in effect sizes across studies. The test of between-subgroup homogeneity yielded a Q-value of 3.72 (*p* = 0.160), suggesting that the differences in effect sizes between intervention types were not statistically significant.

**Figure 5:**
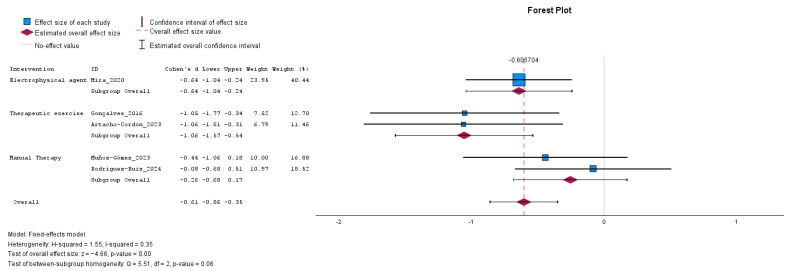
Forest plot presenting the effects of rehabilitative interventions on the improvement of quality of life measured with the Endometriosis-health profile questionnaire in women with endometriosis compared with control; pre-post intervention data. Values on *x*-axis denote Cohen’s d. The diamond illustrates the 95% confidence interval of the pooled effects [27,28,31,35,36].

A total of five studies evaluating the effect of rehabilitative interventions on QoL were included in the meta-analysis (Figure 5). The overall effect size indicated a significant improvement in QoL in favor of the intervention group, with a mean difference of −0.61 (95% CI: from −0.86 to −0.35; *p* < 0.001). The effect was statistically significant for interventions based on electrophysical agents [−0.64 (95% CI: from −1.04 to −0.24; *p* = 0.002)], although this estimate was derived from a single study. A significant effect was also observed for therapeutic exercise [−1.06 (95% CI: from −1.57 to −0.54; *p* < 0.001)], while the effect of manual therapy was not statistically significant [−0.26 (95% CI: from −0.68 to 0.17; *p* = 0.242]. The analysis revealed moderate heterogeneity among the included studies (H^2^ = 1.55, I^2^ = 35%), and a fixed-effect model was applied. The test of between-subgroup homogeneity yielded a Q-value of 5.51 with 2 degrees of freedom (*p* = 0.060), suggesting a trend toward statistically significant differences between intervention types.

## 4. Discussion

This review shows that a variety of physical rehabilitation approaches have shown promising effects in the management of symptoms related to endometriosis and adenomyosis. Overall, most of the interventions evaluated demonstrate beneficial effects on key clinical outcomes such as pain, QoL, or mental health. These interventions include electrophysical agents (e.g., TENS, NMES, CRMRF, RAVANS, and laser therapy), therapeutic exercise, PFP, and manual therapy, among others. Pain-related outcomes were the most frequently assessed, with most studies reporting significant reductions compared to control groups. Similarly, QoL—evaluated in ten out of seventeen trials (58.8%)—improved in nine of them after intervention. Anxiety and depression were assessed in five trials, with improvements observed in 80% and 40% of the trials, respectively. Additionally, a smaller number of trials explored the effects of physical rehabilitation on musculoskeletal impairments (e.g., lumbopelvic muscle function), as well as on sexual and gastrointestinal function. Other benefits reported in individual trials assessing the impact of therapeutic exercise included improvements in fatigue, cardiorespiratory fitness, total testosterone levels, and the prevention of bone mineral density loss in the femoral neck.

Endometriosis-related symptoms have traditionally been managed using medical and/or surgical treatments. However, the persistence of symptom burden despite these approaches has been widely reported [45], highlighting the need for complementary strategies to enhance symptom control. In this context, combining conventional treatments with adjuvant therapies—such as physical rehabilitation—may offer a more comprehensive approach. The mechanisms underlying pelvic pain in endometriosis are complex and multifactorial. In addition to nociceptive pain caused by the presence of ectopic endometrial tissue and associated inflammation, many patients develop central sensitization, characterized by heightened pain perception and altered pain modulation. Moreover, musculoskeletal dysfunctions—such as pelvic floor hypertonicity, myofascial trigger points, and impaired lumbopelvic stability—are frequently observed in this population and may contribute to the persistence and amplification of pain. Physical rehabilitation interventions, including therapeutic exercise, manual therapy, and pelvic floor physiotherapy, may help modulate these mechanisms by improving muscle function, reducing myofascial tension, enhancing neuromuscular control, and promoting endogenous pain inhibition [46,47]. These effects may explain the observed improvements in pain and related outcomes across the included studies. At the time of its publication, the 2022 ESHRE guideline concluded that no specific recommendation could be made for non-medical interventions, including physiotherapy, electrotherapy, exercise, or psychological therapies, due to the limited strength of available evidence at that time [48]. However, several trials published since then have contributed new data supporting the potential benefits of these approaches in women with endometriosis. Psychological interventions, for instance, have shown efficacy in alleviating endometriosis-related pain and mental health symptoms [49,50], although their impact on other clinical outcomes appears to be limited. In contrast, physical rehabilitation approaches may offer a more holistic strategy for managing the multifaceted symptom burden of endometriosis. In addition to improvements in pain and QoL—assessed in 76.5% and 58.8% of the included trials, respectively—several physical rehabilitation interventions demonstrated significant benefits in both local and systemic musculoskeletal impairments, including pelvic floor muscle contractility [26], lumbopelvic muscle thickness, strength, stability [36], and mobility [31], as well as in cardiorespiratory fitness and fatigue [44]. Moreover, some interventions have reported positive effects on sexual and gastrointestinal function [27,44], sleep quality [35,44], hormone levels [40], and bone mineral density in the femoral neck [39]. However, these findings should be interpreted with caution due to the heterogeneity of interventions, the presence of methodological limitations, and the limited reproducibility across studies.

Moreover, given the fluctuations in symptom severity across different phases of the menstrual cycle in women with endometriosis and adenomyosis, it is important to consider how interventions may influence these variations. Only a few trials have specifically addressed—and confirmed—the impact of interventions on dysmenorrhea [26,35,36], while other menstrual-related outcomes remain largely unexplored. In this regard, some authors attempted to standardize outcome measurements by collecting data on the same days of the menstrual cycle to minimize the influence of hormonal fluctuations on symptom reporting [35,36]. Additionally, none of the included studies examined the effectiveness of interventions across different stages of endometriosis, as defined by the American Society for Reproductive Medicine (ASRM), or according to the anatomical location of endometriotic lesions. Given the clinical heterogeneity of these conditions, future research should aim to identify the subgroups of patients who are more likely to benefit from specific rehabilitation interventions.

This systematic review identifies several gaps in the current body of knowledge. Firstly, although pain has been widely addressed, pain sensations in body regions beyond the lumbopelvic area—such as the temporomandibular joint—or signs of central sensitization have been scarcely evaluated [36]. Furthermore, endometriosis has recently been conceptualized as a condition that extends beyond pain, with a high prevalence of severe fatigue and global physical deconditioning [17,51]. However, these aspects have only been addressed in a single trial, which demonstrated the benefits of multimodal therapeutic exercise for these impairments [44]. Regarding mental health, apart from anxiety and depression, other psychological symptoms and disease-related side effects have received little to no attention in the included studies. Similarly, the impact of rehabilitation interventions on occupational performance limitations—often resulting from the physical and psychological burden of endometriosis [19,20,52]—has not been explored. This review also reveals that the efficacy of many potentially beneficial rehabilitation techniques has not yet been tested in this patient population. Physical rehabilitation encompasses a wide range of modalities that could be effective in managing both physical and psychosocial impairments associated with endometriosis and adenomyosis. While some forms of therapeutic exercise and electrophysical agents have been evaluated, other promising interventions remain unexplored. These include aquatic exercise, dry needling, myofascial induction therapy, Kinesio taping, musculoskeletal manipulations (e.g., mobilization and motion therapy), and multimodal occupational therapy interventions incorporating equipment adaptation, environmental modifications, and energy conservation strategies. Moreover, considering the known placebo effects in chronic pain populations, future studies should prioritize blinding when feasible. In this review, five trials evaluating electrophysical agents employed patient-blinded designs by using deactivated devices. However, blinding is more challenging in interventions such as therapeutic exercise. Nonetheless, placebo effects should not be dismissed, as they are clinically relevant and ethically acceptable tools for alleviating chronic pain [53]. In fact, a supportive patient–clinician relationship may enhance these effects, contributing to improved outcomes in individuals with chronic pain [53].

Overall, the significant improvements reported across multiple trials, combined with the diversity of therapeutic approaches —including electrophysical agents, therapeutic exercise, and manual techniques— make it difficult to determine the single most effective rehabilitation strategy for managing endometriosis-related symptoms. Most interventions demonstrated benefits in pain reduction and improvements in QoL; however, other relevant outcomes were not consistently assessed across all modalities. For instance, no evidence was found regarding the effectiveness of electrophysical agents on musculoskeletal impairments. Nevertheless, considering the broader range of effects associated with each therapeutic approach, and based on the findings summarized in this review, therapeutic exercise programs appear to offer the most comprehensive benefits. These not only include improvements in pain and QoL, but also positive effects on musculoskeletal function and mental health.

The findings of this systematic review are consistent with several meta-analyses that have examined the effects of various physical rehabilitation approaches in chronic pain conditions [54,55,56], and particularly in female-specific disorders [57,58,59], all of which reported favorable outcomes for the intervention groups. Moreover, our results align with a previous meta-analysis that included six trials evaluating the effectiveness of physical therapy in women with endometriosis, specifically for improving pain and QoL [60]. However, that study did not include trials assessing other relevant outcomes. Additionally, a systematic review published in 2018 focused exclusively on therapeutic exercise [61], although only two trials were available at that time. In contrast, the present review incorporates those earlier studies along with more recent trials, providing a more comprehensive and updated overview of the available scientific evidence on physical rehabilitation interventions for endometriosis and adenomyosis.

Although this systematic review has encompassed a wide range of physical rehabilitation interventions, the conclusions regarding their effectiveness in women with endometriosis and/or adenomyosis remain weak. This is primarily due to the heterogeneity of the proposed interventions —many of which were not replicated across studies— and the diversity of outcome measures, as well as the high risk of bias identified in numerous studies. Nevertheless, taken together, the findings suggest that women with endometriosis and/or adenomyosis may benefit from rehabilitative interventions, as all included studies reported improvements in at least one disease-related outcome. These results underscore the need for well-designed randomized controlled trials to rigorously evaluate the effectiveness of physical rehabilitation in managing the common symptoms associated with endometriosis and adenomyosis.

### Study Limitations

This systematic review and meta-analysis has several limitations. First, the included studies showed considerable variability in the diagnostic criteria used to confirm endometriosis or adenomyosis, and many did not report the disease stage. Such heterogeneity may limit the internal validity and comparability of the findings. Second, it was not possible to pool data from some studies due to methodological heterogeneity, which limited the scope of the meta-analysis. Third, although the literature search was conducted using the three major public health databases, relevant publications indexed exclusively in other databases may have been missed. Fourth, the review was restricted to controlled trials, thereby excluding potentially informative evidence from non-controlled studies. Finally, the studies included in the meta-analysis exhibited considerable clinical heterogeneity in terms of the interventions assessed, which may affect the generalizability of the findings.

## 5. Conclusions

This systematic review and meta-analysis identifies the promising benefits of physical rehabilitation for patients with endometriosis. Various approaches—including electrophysical agents, therapeutic exercise, and manual therapy techniques—appear to improve certain clinical outcomes related to endometriosis, particularly pain and QoL. However, the lack of replication of interventions across studies and the overall high risk of bias in the available literature limit the ability to identify the most effective strategies for symptom management. Therefore, further well-designed randomized controlled trials are urgently needed to confirm these preliminary findings and to evaluate the effectiveness of additional therapeutic approaches, while also expanding the assessment to other relevant outcomes not previously explored. Regarding adenomyosis, no controlled trials were identified that assess the effectiveness of rehabilitation interventions in this population, highlighting a critical gap and the need for future research targeting this subgroup of patients.

## Figures and Tables

**Table 3:** Summary of the results, their certainty, and clinical relevance assessed using the GRADE methodology.

Certainty Assessment							No. of Patients		Effect	Certainty
No. of Studies	Study Design	Risk of Bias	Inconsistency	Indirectness	Imprecision	Other Considerations	Intervention	Control	Absolute (95% CI)	
**PAIN INTENSITY (assessed with: NRS/VAS; Scale from: 0 to 10 (worse))**										
Pain intensity in women with endometriosis after rehabilitation treatment compared with placebo/usual care treatment										
11	Randomized trials	Serious ^a^	Serious ^b^	Not serious	Not serious	None	325	284	SMD 1.19 SD lower (1.72 lower to 0.67 lower)	⨁⨁◯◯ Low ^a,b^
Pain intensity in women with endometriosis after electrophysical agent treatment compared with placebo/usual care treatment										
5	Randomized trials	Serious ^a^	Not serious	Not serious	Not serious	None	215	197	SMD 1.43 SD lower (2.01 lower to 0.86 lower)	⨁⨁⨁◯ Moderate ^a^
Pain intensity in women with endometriosis after therapeutic exercise treatment compared with placebo/usual care treatment										
3	Randomized trials	Serious ^a^	Serious ^b^	Not serious	Serious ^c^	None	51	31	SMD 1.46 SD lower (3.16 lower to 0.24 higher)	⨁◯◯◯ Very low ^a,b,c^
Pain intensity in women with endometriosis after manual therapy treatment compared with placebo/usual care treatment										
3	Randomized trials	Serious ^a^	Serious ^b^	Not serious	Not serious	None	59	56	SMD 0.54 SD lower (1.26 lower to 0.18 higher)	⨁⨁◯◯ Low ^a,b^
**QUALITY OF LIFE (assessed with: EHP; Scale from: 0 to 100 (worse))**										
Quality of life in women with endometriosis after rehabilitation treatment compared with placebo/usual care treatment										
5	Randomized trials	Serious ^a^	Not serious	Not serious	Not serious	None	139	118	SMD 0.61 SD lower (0.94 lower to 0.29 lower)	⨁⨁⨁◯ Moderate ^a^
Quality of life in women with endometriosis after electrophysical agent treatment compared with placebo/usual care treatment										
1	Randomized trials	Serious ^a^	Serious ^b^	Not serious	Not serious	None	53	48	SMD 0.64 SD lower (1.04 lower to 0.24 lower)	⨁⨁◯◯ Low ^a,b^
Quality of life in women with endometriosis after therapeutic exercise treatment compared with placebo/usual care treatment										
2	Randomized trials	Serious ^a^	Not serious	Not serious	Serious ^c^	None	44	27	SMD 1.06 SD lower (1.57 lower to 0.54 lower)	⨁⨁◯◯ Low ^a,c^
Quality of life in women with endometriosis after manual therapy treatment compared with placebo/usual care treatment										
2	Randomized trials	Serious ^a^	Serious ^b^	Not serious	Serious ^c^	None	42	43	SMD 0.26 SD lower (0.68 lower to 0.17 higher)	⨁◯◯◯ Very low ^a,b,c^

CI: confidence interval; EHP-30: Endometriosis Health Profile-30; NRS: numeric rating scale; SD: standard deviation, SMD: standardized mean difference; VAS: visual analogue scale. ^a^ Lack of blinding of both participants and clinicians administering the intervention, which may have introduced performance and detection bias. ^b^ Substantial heterogeneity across studies without a clear explanation. ^c^ Wide confidence intervals. ⨁⨁◯◯: Low; ⨁⨁⨁◯: Moderate; ⨁◯◯◯: Very Low.

## Supplementary Materials

The following supporting information can be downloaded at: https://www.mdpi.com/article/10.3390/jcm14238284/s1, Table S1: Intervention parameters on the included randomized controlled trials.

## Appendix A. Search Strategy

### Appendix A.1. Medline

**Table:**

Date	23 July 2025
Strategy	#1 AND #2 AND #3
**#1**	(((((((endometriosis[MeSH Terms]) OR (adenomyosis[MeSH Terms])) OR (endometriosis[Title/Abstract])) OR (endometrioses[Title/Abstract])) OR (endometrioma[Title/Abstract])) OR (adenomyosis[Title/Abstract])) OR (endometriomas[Title/Abstract]))
**#2**	(((((((((((((((((((((((((((((((((((((((((((((((((((((((((((((((((((((((((((((((((((((((((((((((((((((((((((((((((((((((((((((((Myofunctional Therapy[MeSH Terms]) OR (occupational therapy[MeSH Terms])) OR (physical therapy modalities[MeSH Terms])) OR (resistance training[MeSH Terms])) OR (plyometric exercise[MeSH Terms])) OR (breathing exercises[MeSH Terms])) OR (Exercise[MeSH Terms])) OR (exercise movement techniques[MeSH Terms])) OR (yoga[MeSH Terms])) OR (muscle stretching exercises[MeSH Terms])) OR (motion therapy, continuous passive[MeSH Terms])) OR (Endurance training[MeSH Terms])) OR (exercise therapy[MeSH Terms])) OR (activities of daily living[MeSH Terms])) OR (rehabilitation[MeSH Terms])) OR (dance therapy[MeSH Terms])) OR (recreation therapy[MeSH Terms])) OR (tai ji[MeSH Terms])) OR (electric stimulation therapy[MeSH Terms])) OR (hydrotherapy[MeSH Terms])) OR (“Myofunctional Therapy”[Title/Abstract])) OR (“Myofunctional Therapies”[Title/Abstract])) OR (“Occupational Therapy”[Title/Abstract])) OR (“Occupational Therapies”[Title/Abstract])) OR (“Physical Therapy Modalities”[Title/Abstract])) OR (“Physical Therapy Modality”[Title/Abstract])) OR (“Physical Therapy”[Title/Abstract])) OR (Physiotherapy[Title/Abstract])) OR (Physiotherapies[Title/Abstract])) OR (“Physical Therapy Techniques”[Title/Abstract])) OR (“Physical Therapy Technique”[Title/Abstract])) OR (“Electric Stimulation Therapy”[Title/Abstract])) OR (“Therapeutic Electrical Stimulation”[Title/Abstract])) OR (“Therapeutic Electric Stimulation”[Title/Abstract])) OR (“Electrical Stimulation Therapy”[Title/Abstract])) OR (Electrotherapy[Title/Abstract])) OR (“laser”[Title/Abstract]) OR (“manual therapy”[Title/Abstract]) OR (“TENS”[Title/Abstract]) OR (“NMES”[Title/Abstract]) OR (“muscular relaxation training”[Title/Abstract]) OR (“physical training”[Title/Abstract]) OR (“massage”[Title/Abstract]) OR (“virtual reality”[Title/Abstract]) OR (“Resistance Training”[Title/Abstract])) OR (“Weight-Lifting Exercise Program”[Title/Abstract])) OR (“Weight Lifting Exercise Program”[Title/Abstract])) OR (“Weight-Lifting Exercise Programs”[Title/Abstract])) OR (“Weight-Bearing Strengthening Program”[Title/Abstract])) OR (“Strengthening Program”[Title/Abstract])) OR (“Strengthening Programs”[Title/Abstract])) OR (“Exercise Program”[Title/Abstract])) OR (“Exercise Programs”[Title/Abstract])) OR (“Weight Bearing Strengthening Program”[Title/Abstract])) OR (“Weight-Bearing Strengthening Programs”[Title/Abstract])) OR (“Weight-Bearing Exercise Program”[Title/Abstract])) OR (“Weight Bearing Exercise Program”[Title/Abstract])) OR (“Weight-Bearing Exercise Programs”[Title/Abstract])) OR (“Plyometric Exercise”[Title/Abstract])) OR (“Plyometric Exercises”[Title/Abstract])) OR (“Plyometric Drill”[Title/Abstract])) OR (“Plyometric Drills”[Title/Abstract])) OR (“Plyometric Training”[Title/Abstract])) OR (“Plyometric Trainings”[Title/Abstract])) OR (“Stretch-Shortening Exercise”[Title/Abstract])) OR (“Stretch Shortening Exercise”[Title/Abstract])) OR (“Stretch-Shortening Exercises”[Title/Abstract])) OR (“Stretch-Shortening Cycle Exercise”[Title/Abstract])) OR (“Stretch Shortening Cycle Exercise”[Title/Abstract])) OR (“Stretch-Shortening Cycle Exercises”[Title/Abstract])) OR (“Stretch-Shortening Drills”[Title/Abstract])) OR (“Breathing Exercises”[Title/Abstract])) OR (“Physical Activity”[Title/Abstract])) OR (“Physical Activities”[Title/Abstract])) OR (“Physical Exercise”[Title/Abstract])) OR (“Physical Exercises”[Title/Abstract])) OR (“Acute Exercise”[Title/Abstract])) OR (“Acute Exercises”[Title/Abstract])) OR (“Isometric Exercises”[Title/Abstract])) OR (“Isometric Exercise”[Title/Abstract])) OR (“Aerobic Exercise”[Title/Abstract])) OR (“Aerobic Exercises”[Title/Abstract])) OR (“Exercise Training”[Title/Abstract])) OR (“Exercise Trainings”[Title/Abstract])) OR (“Exercise Movement Techniques”[Title/Abstract])) OR (“Exercise Movement Technics”[Title/Abstract])) OR (“Pilates-Based Exercises”[Title/Abstract])) OR (“Pilates Based Exercises”[Title/Abstract])) OR (“Pilates Training”[Title/Abstract])) OR (yoga[Title/Abstract])) OR (“Muscle Stretching Exercises”[Title/Abstract])) OR (“Active Stretching”[Title/Abstract])) OR (“Muscle Stretching Exercise”[Title/Abstract])) OR (“Static Stretching”[Title/Abstract])) OR (“Static-Active Stretching”[Title/Abstract])) OR (“Static Active Stretching”[Title/Abstract])) OR (“Isometric Stretching”[Title/Abstract])) OR (“Dynamic Stretching”[Title/Abstract])) OR (“Proprioceptive Neuromuscular Facilitation”[Title/Abstract])) OR (“Neuromuscular Facilitation”[Title/Abstract])) OR (“Passive Stretching”[Title/Abstract])) OR (“Static-Passive Stretching”[Title/Abstract])) OR (“Static Passive Stretching”[Title/Abstract])) OR (“Continuous Passive Motion Therapy”[Title/Abstract])) OR (“CPM Therapy”[Title/Abstract])) OR (“CPM Therapies”[Title/Abstract])) OR (“Endurance Training”[Title/Abstract])) OR (“Exercise Therapy”[Title/Abstract])) OR (“Rehabilitation Exercise”[Title/Abstract])) OR (“Rehabilitation Exercises”[Title/Abstract])) OR (“Activities of Daily Living”[Title/Abstract])) OR (“Daily Living Activities”[Title/Abstract])) OR (“Daily Living Activity”[Title/Abstract])) OR (“Chronic Limitation of Activity”[Title/Abstract])) OR (“Dance Therapy”[Title/Abstract])) OR (“Dance Therapies”[Title/Abstract])) OR (“Recreation Therapy”[Title/Abstract])) OR (“Recreational Therapy”[Title/Abstract])) OR (“Recreational Therapies”[Title/Abstract])) OR (Rehabilitation[Title/Abstract])) OR (habilitation[Title/Abstract])) OR (ADL[Title/Abstract])) OR (“Remedial Exercise”[Title/Abstract])) OR (“Remedial Exercises”[Title/Abstract])) OR (“Ballistic Stretching”[Title/Abstract])) OR (“Proprioceptive Neuromuscular Facilitation Stretching”[Title/Abstract])) OR (“Exercise Programme”[Title/Abstract]))
**#3**	((((((((((((Randomized Controlled Trials as Topic[MeSH Terms])) OR (Clinical Studies as Topic[MeSH Terms])) OR (Controlled Clinical Trials as Topic[MeSH Terms])) OR (Controlled Clinical Trial[Publication Type])) OR (Randomized Controlled Trial[Publication Type])) OR (Clinical Trial[Publication Type])) OR (“Randomized Controlled Trial”[Title/Abstract])) OR (“Controlled Clinical Trial”[Title/Abstract])) OR (“randomized controlled clinical trial”[Title/Abstract])) OR (“randomised controlled clinical trial”[Title/Abstract])) OR (“randomised controlled trial”[Title/Abstract]))

### Appendix A.2. Web of Science

**Table:**

Date	23 July 2025
Strategy	#1 AND #2 AND #3
**#1**	TS = (endometriosis OR adenomyosis OR endometrioses OR endometrioma OR endometriomas)
**#2**	TS = (“Myofunctional Therapy” OR “Myofunctional Therapies” OR “Occupational Therapy” OR “Occupational Therapies” OR “Physical Therapy Modalities” OR “Physical Therapy Modality” OR “Physical Therapy” OR “physiotherapy” OR “physiotherapies” OR “Physical Therapy Techniques” OR “Physical Therapy Technique” OR “Electric Stimulation Therapy” OR “Therapeutic Electrical Stimulation” OR “Therapeutic Electric Stimulation” OR “Electrical Stimulation Therapy” OR “Electrotherapy” OR “Laser” OR “Manual Therapy” OR “TENS” OR “NMES” OR “Muscular Relaxation Training” OR “Physical Training” OR “Massage” OR “Virtual Reality” OR “Resistance Training” OR “Weight-Lifting Exercise Program” OR “Weight Lifting Exercise Program” OR “Weight-Lifting Exercise Programs” OR “Weight-Bearing Strengthening Program” OR “Strengthening Program” OR “Strengthening Programs” OR “Exercise Program” OR “Exercise Programs” OR “Weight Bearing Strengthening Program” OR “Weight-Bearing Strengthening Programs” OR “Weight-Bearing Exercise Program” OR “Weight Bearing Exercise Program” OR “Weight-Bearing Exercise Programs” OR “Plyometric Exercise” OR “Plyometric Exercises” OR “Plyometric Drill” OR “Plyometric Drills” OR “Plyometric Training” OR “Plyometric Trainings” OR “Stretch-Shortening Exercise” OR “Stretch Shortening Exercise” OR “Stretch-Shortening Exercises” OR “Stretch-Shortening Cycle Exercise” OR “Stretch Shortening Cycle Exercise” OR “Stretch-Shortening Cycle Exercises” OR “Stretch-Shortening Drills” OR “Breathing Exercises” OR “Physical Activity” OR “Physical Activities” OR “Physical Exercise” OR “Physical Exercises” OR “Acute Exercise” OR “Acute Exercises” OR “Isometric Exercises” OR “Isometric Exercise” OR “Aerobic Exercise” OR “Aerobic Exercises” OR “Exercise Training” OR “Exercise Trainings” OR “Exercise Movement Techniques” OR “Exercise Movement Technics” OR “Pilates-Based Exercises” OR “Pilates Based Exercises” OR “Pilates Training” OR “yoga” OR “Muscle Stretching Exercises” OR “Active Stretching” OR “Muscle Stretching Exercise” OR “Static Stretching” OR “Static-Active Stretching” OR “Static Active Stretching” OR “Isometric Stretching” OR “Dynamic Stretching” OR “Proprioceptive Neuromuscular Facilitation” OR “Neuromuscular Facilitation” OR “Passive Stretching” OR “Static-Passive Stretching” OR “Static Passive Stretching” OR “Continuous Passive Motion Therapy” OR “CPM Therapy” OR “CPM Therapies” OR “Endurance Training” OR “Exercise Therapy” OR “Rehabilitation Exercise” OR “Rehabilitation Exercises” OR “Activities of Daily Living” OR “Daily Living Activities” OR “Daily Living Activity” OR “Chronic Limitation of Activity” OR “Dance Therapy” OR “Dance Therapies” OR “Recreation Therapy” OR “Recreational Therapy” OR “Recreational Therapies” OR “rehabilitation” OR “habilitation” OR “ADL” OR “Remedial Exercise” OR “Remedial Exercises” OR “Ballistic Stretching” OR “Proprioceptive Neuromuscular Facilitation Stretching” OR “Exercise Programme”)
**#3**	TS = (“Controlled Clinical Trial” OR “Randomized Controlled Trial” OR “Clinical Trial” OR “Randomized Controlled Trial” OR “Controlled Clinical Trial” OR “randomized controlled clinical trial” OR “randomised controlled clinical trial” OR “randomised controlled trial”)

### Appendix A.3. Scopus

**Table:**

Date	23 July 2025
Strategy	#1 AND #2 AND #3
**#1**	((TITLE-ABS-KEY (endometriosis) OR TITLE-ABS-KEY (adenomyosis) OR TITLE-ABS-KEY (endometrioses) OR TITLE-ABS-KEY (endometrioma) OR TITLE-ABS-KEY (endometriomas)))
**#2**	((TITLE-ABS-KEY (“Myofunctional Therapy”) OR TITLE-ABS-KEY (“Myofunctional Therapies”) OR TITLE-ABS-KEY (“Occupational Therapy”) OR TITLE-ABS-KEY (“Occupational Therapies”) OR TITLE-ABS-KEY (“Physical Therapy Modalities”) OR TITLE-ABS-KEY (“Physical Therapy Modality”) OR TITLE-ABS-KEY (“Physical Therapy”) OR TITLE-ABS-KEY (“physiotherapy”) OR TITLE-ABS-KEY (“physiotherapies”) OR TITLE-ABS-KEY (“Physical Therapy Techniques”) OR TITLE-ABS-KEY (“Physical Therapy Technique”) OR TITLE-ABS-KEY (“Electric Stimulation Therapy”) OR TITLE-ABS-KEY (“Therapeutic Electrical Stimulation”) OR TITLE-ABS-KEY (“Therapeutic Electric Stimulation”) OR TITLE-ABS-KEY (“Electrical Stimulation Therapy”) OR TITLE-ABS-KEY (“Electrotherapy”) OR TITLE-ABS-KEY (“Laser”) OR TITLE-ABS-KEY (“Manual Therapy”) OR TITLE-ABS-KEY (“TENS”) OR TITLE-ABS-KEY (“NMES”) OR TITLE-ABS-KEY (“Muscular Relaxation Training”) OR TITLE-ABS-KEY (“Physical Training”) OR TITLE-ABS-KEY (“Massage”) OR TITLE-ABS-KEY (“Virtual Reality”) OR TITLE-ABS-KEY (“Resistance Training”) OR TITLE-ABS-KEY (“Weight-Lifting Exercise Program”) OR TITLE-ABS-KEY (“Weight Lifting Exercise Program”) OR TITLE-ABS-KEY (“Weight-Lifting Exercise Programs”) OR TITLE-ABS-KEY (“Weight-Bearing Strengthening Program”) OR TITLE-ABS-KEY (“Strengthening Program”) OR TITLE-ABS-KEY (“Strengthening Programs”) OR TITLE-ABS-KEY (“Exercise Program”) OR TITLE-ABS-KEY (“Exercise Programs”) OR TITLE-ABS-KEY (“Weight Bearing Strengthening Program”) OR TITLE-ABS-KEY (“Weight-Bearing Strengthening Programs”) OR TITLE-ABS-KEY (“Weight-Bearing Exercise Program”) OR TITLE-ABS-KEY (“Weight Bearing Exercise Program”) OR TITLE-ABS-KEY (“Weight-Bearing Exercise Programs”) OR TITLE-ABS-KEY (“Plyometric Exercise”) OR TITLE-ABS-KEY (“Plyometric Exercises”) OR TITLE-ABS-KEY (“Plyometric Drill”) OR TITLE-ABS-KEY (“Plyometric Drills”) OR TITLE-ABS-KEY (“Plyometric Training”) OR TITLE-ABS-KEY (“Plyometric Trainings”) OR TITLE-ABS-KEY (“Stretch-Shortening Exercise”) OR TITLE-ABS-KEY (“Stretch Shortening Exercise”) OR TITLE-ABS-KEY (“Stretch-Shortening Exercises”) OR TITLE-ABS-KEY (“Stretch-Shortening Cycle Exercise”) OR TITLE-ABS-KEY (“Stretch Shortening Cycle Exercise”) OR TITLE-ABS-KEY (“Stretch-Shortening Cycle Exercises”) OR TITLE-ABS-KEY (“Stretch-Shortening Drills”) OR TITLE-ABS-KEY (“Breathing Exercises”) OR TITLE-ABS-KEY (“Physical Activity”) OR TITLE-ABS-KEY (“Physical Activities”) OR TITLE-ABS-KEY (“Physical Exercise”) OR TITLE-ABS-KEY (“Physical Exercises”) OR TITLE-ABS-KEY (“Acute Exercise”) OR TITLE-ABS-KEY (“Acute Exercises”) OR TITLE-ABS-KEY (“Isometric Exercises”) OR TITLE-ABS-KEY (“Isometric Exercise”) OR TITLE-ABS-KEY (“Aerobic Exercise”) OR TITLE-ABS-KEY (“Aerobic Exercises”) OR TITLE-ABS-KEY (“Exercise Training”) OR TITLE-ABS-KEY (“Exercise Trainings”) OR TITLE-ABS-KEY (“Exercise Movement Techniques”) OR TITLE-ABS-KEY (“Exercise Movement Technics”) OR TITLE-ABS-KEY (“Pilates-Based Exercises”) OR TITLE-ABS-KEY (“Pilates Based Exercises”) OR TITLE-ABS-KEY (“Pilates Training”) OR TITLE-ABS-KEY (“ yoga”) OR TITLE-ABS-KEY (“Muscle Stretching Exercises”) OR TITLE-ABS-KEY (“Active Stretching”) OR TITLE-ABS-KEY (“Muscle Stretching Exercise”) OR TITLE-ABS-KEY (“Static Stretching”) OR TITLE-ABS-KEY (“Static-Active Stretching”) OR TITLE-ABS-KEY (“Static Active Stretching”) OR TITLE-ABS-KEY (“Isometric Stretching”) OR TITLE-ABS-KEY (“Dynamic Stretching”) OR TITLE-ABS-KEY (“Proprioceptive Neuromuscular Facilitation”) OR TITLE-ABS-KEY (“Neuromuscular Facilitation”) OR TITLE-ABS-KEY (“Passive Stretching”) OR TITLE-ABS-KEY (“Static-Passive Stretching”) OR TITLE-ABS-KEY (“Static Passive Stretching”) OR TITLE-ABS-KEY (“Continuous Passive Motion Therapy”) OR TITLE-ABS-KEY (“CPM Therapy”) OR TITLE-ABS-KEY (“CPM Therapies”) OR TITLE-ABS-KEY (“Endurance Training”) OR TITLE-ABS-KEY (“Exercise Therapy”) OR TITLE-ABS-KEY (“Rehabilitation Exercise”) OR TITLE-ABS-KEY (“Rehabilitation Exercises”) OR TITLE-ABS-KEY (“Activities of Daily Living”) OR TITLE-ABS-KEY (“Daily Living Activities”) OR TITLE-ABS-KEY (“Daily Living Activity”) OR TITLE-ABS-KEY (“Chronic Limitation of Activity”) OR TITLE-ABS-KEY (“Dance Therapy”) OR TITLE-ABS-KEY (“Dance Therapies”) OR TITLE-ABS-KEY (“Recreation Therapy”) OR TITLE-ABS-KEY (“Recreational Therapy”) OR TITLE-ABS-KEY (“Recreational Therapies”) OR TITLE-ABS-KEY (“rehabilitation”) OR TITLE-ABS-KEY (“habilitation”) OR TITLE-ABS-KEY (“adl”) OR TITLE-ABS-KEY (“Remedial Exercise”) OR TITLE-ABS-KEY (“Remedial Exercises”) OR TITLE-ABS-KEY (“Ballistic Stretching”) OR TITLE-ABS-KEY (“Proprioceptive Neuromuscular Facilitation Stretching”) OR TITLE-ABS-KEY (“Exercise Programme”)))
**#3**	((TITLE-ABS-KEY (“Controlled Clinical Trial”) OR TITLE-ABS-KEY (“Randomized Controlled Trial”) OR TITLE-ABS-KEY (“Clinical Trial”) OR TITLE-ABS-KEY (“Randomized Controlled Trial”) OR TITLE-ABS-KEY (“Controlled Clinical Trial”) OR TITLE-ABS-KEY (“randomized controlled clinical trial”) OR TITLE-ABS-KEY (“randomised controlled clinical trial”) OR TITLE-ABS-KEY (“randomised controlled trial”)))
